# Overexpression of *SlPRE2*, an atypical bHLH transcription factor, affects plant morphology and fruit pigment accumulation in tomato

**DOI:** 10.1038/s41598-017-04092-y

**Published:** 2017-07-19

**Authors:** Zhiguo Zhu, Guoping Chen, Xuhu Guo, Wencheng Yin, Xiaohui Yu, Jingtao Hu, Zongli Hu

**Affiliations:** Laboratory of molecular biology of tomato, Bioengineering College, Chongqing University, Chongqing, 400044 People’s Republic of China

## Abstract

The basic helix-loop-helix (bHLH) proteins are a large family of transcription factors that control various developmental processes in eukaryotes, but the biological roles of most bHLH proteins are not very clear, especially in tomato. In this study, a *PRE*-like atypical bHLH gene was isolated and designated as *SlPRE2* in tomato. *SlPRE2* was highly expressed in immature-green fruits, moderately in young leaves, flowers, and mature-green fruits. To further research the function of *SlPRE2*, a *35 S:PRE2* binary vector was constructed and transformed into wild type tomato. The transgenic plants showed increased leaf angle and stem internode length, rolling leaves with decreased chlorophyll content. The water loss rate of detached leaves was increased in young transgenic lines but depressed in mature leaves. Besides, overexpression of *SlPRE2* promoted morphogenesis in seedling development, producing light-green unripening fruits and yellowing ripen fruits with reduced chlorophyll and carotenoid accumulation in pericarps, respectively. Quantitative RT-PCR analysis showed that expression of the chlorophyll related genes, such as *GOLDEN 2-LIKE* and *RbcS*, were decreased in unripening *35* *S:PRE2* fruit, and carotenoid biosynthesis-related genes *PHYTOENE SYNTHASE1A* and *ζ-CAROTENE DESATURASE* in ripening fruit were also down-regulated. These results suggest that *SlPRE2* affects plant morphology and is a negative regulator of fruit pigment accumulation.

## Introduction

The basic helix-loop-helix (bHLH) proteins are a large family of transcription factors that control metabolic, physiological and developmental processes in all eukaryotic organisms. They were considered to have different function based on the distinction of bHLH domain^1, 2^. The bHLH protein consists of about 60 amino acids organized in the basic region and HLH region^3^. The HLH region at the C-terminal is involved in the homo- or hetero-dimerization with other protein while the basic region in N-terminal for DNA-binding. Based on the DNA-binding ability, the bHLH proteins are divided into two groups, “DNA-binding bHLH” and “atypical bHLH” with no DNA-binding ability^1, 4^. Recent studies have shown that typical bHLH transcription factors participate in various plant growth and development processes, such as light signaling^5^, hormone signaling^6^, anthocyanin biosynthesis^7, 8^, fruit development^9^ and stress responses^10^. For example, overexpression of *PIF5* induces leaf senescence and chlorophyll degradation in dark-grown *Arabidopsis* ^11^. The *SlPIF1a* is characterized to regulate carotenoid biosynthesis during fruit ripening by a light-dependent mechanism in tomato^12^. A *bHLH35* gene from *populous euphratica* could regulate photosynthesis in *Arabidopsis* ^13^. In addition, the atypical bHLH genes have been shown to play a role in the regulation of hormone signaling^14, 15^, light signaling^16^, vascular development^17^ and grain size^18^, such as *PRE1* and *KIDARI/PRE6* ^19, 20^.

*PRE1* and *KIDARI* belong to the *paclobutrazol-resistant (PRE*) family with 6 members in *Arabidopsis*. *PRE1*, one of these atypical bHLHs, is initially identified to be an activator of gibberellin responses^19^. The further researches show that *PRE1* mediates brassinosteroid, auxin, and light signaling^21–23^. The *PRE3* is a dominant suppressor of BR mutant *bri1-301* and is involved in regulation of light signal transduction in *Arabidopsis* ^24, 25^. The *PRE4/BNQ3* also function in light signaling, and *bnq3* mutant have pale-green flower and reduced chlorophyll level^26^. KIDARI/PRE6 is a repressor of light signaling and affects photomorphogenesis by negatively regulating HFR1 activity^20, 27^. Overexpression of *ILI1* (*INCREASED LAMINA INCLINATION1)*, a homology of *PRE1* in rice, increased cell elongation through a mechanism involved in brassinosteroid signalling^21^. *BU1* (*BRASSINOSTEROID UPREGULATED1*) is involved in brassinosteroid signaling and controls lamina joint bending in rice^28^. Overexpression of *PGL1* (*POSITIVE REGULATOR OF GRAIN LENGTH 1*) and *PGL2* in lemma/palea could increase grain length and weight in transgenic rice^18, 29^. To date, only *Slstyle2.1*, a *PREs*-like gene in tomato, was identified to control floral style length and contribute to the evolution of self-pollination in cultivated variety^30^. Through our genome-wide analysis of *PRE* family, there are five members in tomato including *SlSTYLE2.1*. However, their biological function in tomato growth is still unknown, which undoubtedly need further study.

Tomato is considered to be one of the best systems to study fleshy fruit ripening. Here, a homologous gene of *Arabidopsis PREs*, which was named *SlPRE2* along the *PRE*-like gene *style2.1* ^30^, was cloned. In this study, overexpression of *SlPRE2* was performed to investigate the role of *SlPRE2* in tomato development. Transgenic tomatoes showed alteration of plant morphology by affecting light signaling and repression of fruit pigment accumulation. Our results indicate that *SlPRE2* affects plant morphogenesis, fruit chlorophyll and carotenoid accumulation probably through influencing the activity of bHLH proteins involved in light signaling.

## Results

### *SlPRE2* isolation and transcription pattern analysis

**Figure 1:**
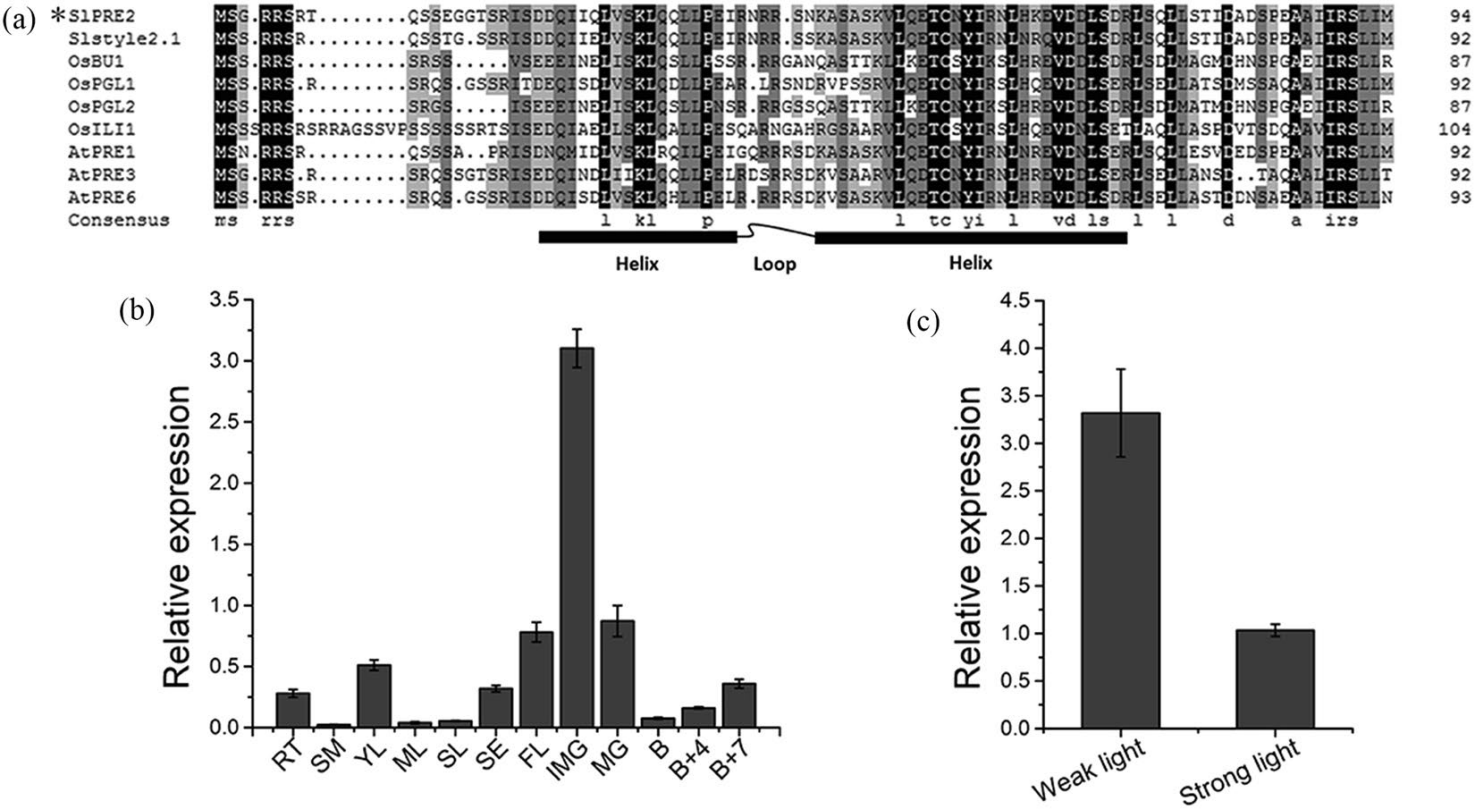
Multiple sequence alignment and expression profile of *SlPRE2*. (**a**) Multiple sequence alignment of SlPRE2 and other bHLH proteins. The SlPRE2 and Slstyle2.1 are proteins from tomato. OsBU1, OsPGL1, OsPGL2, OsILI1 are proteins from rice. *AtPRE1–6* are proteins from *Arabidopsis*. Black and gray backgrounds indicate identical and similar amino acids. Convergence in structure is indicated by black box and curve. (**b**) The relative expression patterns of *SlPRE2* in wild type tomato. RT, root; SM, stem; YL, young leaf; ML, mature leaf; SL, senescent leaf; SE, sepal; FL, flower; IMG, immature green fruit; MG, mature fruit; B, breaker fruit; B + 4, 4 days after breaker stage; B + 7, 7 days after breaker stage. (**c**) The relative expression levels in weak and strong light growth condition for 8 hours. Data are the mean ± SD of three biological replicates.

Based on the BLAST analysis in SGN (Sol Genomics Network, https://solgenomics.net/), 5 AtPREs like genes were isolated and named *SlPRE1* to *SlPRE5* in tomato (Fig. S1a,b). With the transcriptome analysis in SGN, expression profile of these *SlPREs* was performed in Fig. S1c. *SlPRE1*, which has been functionally identified as *STYLE2.1* in tomato^30^, was specifically expressed in anthesis flower. *SlPRE2* was highly expressed in 10 days post anthesis fruits(DPA). *SlPRE3* had low expression abundance. Moreover, *SlPRE4* was highly expressed in hypocotyl and vegetative meristem, while *SlPRE5* was performed expressed in multiple tissue. The *SlPRE2* was chosen for further investigation since its high expression in IMG fruit. Based on the sequence in SGN (sequence ID: Solyc02g067380.2.1), the SlPRE2 was isolated from tomato with specific primers SlPRE2-F and SlPRE2-R. Gene sequence analysis showed that *SlPRE2* encodes a putative bHLH protein consisting of 94 amino acids (Fig. 1a). *SlPRE1* has been functionally identified as *STYLE2.1* in tomato^30^. *SlPRE3* and *SlPRE4* were reported as *SlbHLH103* and *SlbHLH131* by Sun, respectively^31^. As shown in Fig. 1a, amino acid sequence alignment of SlPRE2 and homologous proteins showed that SlPRE2 is highly homologous to AtPREs, OsBU1 and OsPGLs, which were identified as atypical bHLH^18, 20, 23, 24, 28, 29^ (Fig. 1a). These results indicated that *SlPRE2* encodes an atypical bHLH transcription factor with no DNA-binding activity.

To extend our understanding of the role of *SlPRE2* in tomato growth and development, quantitative PCR was performed to analyze the expression of *SlPRE2* in various tissues, including roots, stems, leaves, flowers and fruits at different stages of development, from tomato cultivar Ailsa Craig (AC). The results showed that *SlPRE2* was highly expressed in 15 DPA IMG fruits, moderately in young leaves, flowers, and mature-green fruits, and the lowest expression level was found in stems, mature and senescent leaves (Fig. 1b). Besides, a similar expression pattern of *SlPRE2* in *Solanum pimpinellifolium* LA1589 was performed using Tomato Functional Genomics Database (http://ted.bti.cornell.edu/) (Table S1). Promoter analysis of *SlPRE2* gene demonstrated that there are various light response elements, such as G-Box and ACE motif, located 800 bases upstream of the translation start site (ATG) (Table S2). To further explore its role in light signaling, tomato leaves exposed in weak (50 μmol·m^−2^·s^−1^) or strong light (800 μmol·m^−2^·s^−1^) for 8 hours were collected. Significantly suppressed expression of *SlPRE2* was observed in strong light condition (Fig. 1c).

### Overexpression of *SlPRE2* alters leaf and stem morphology

**Figure 2:**
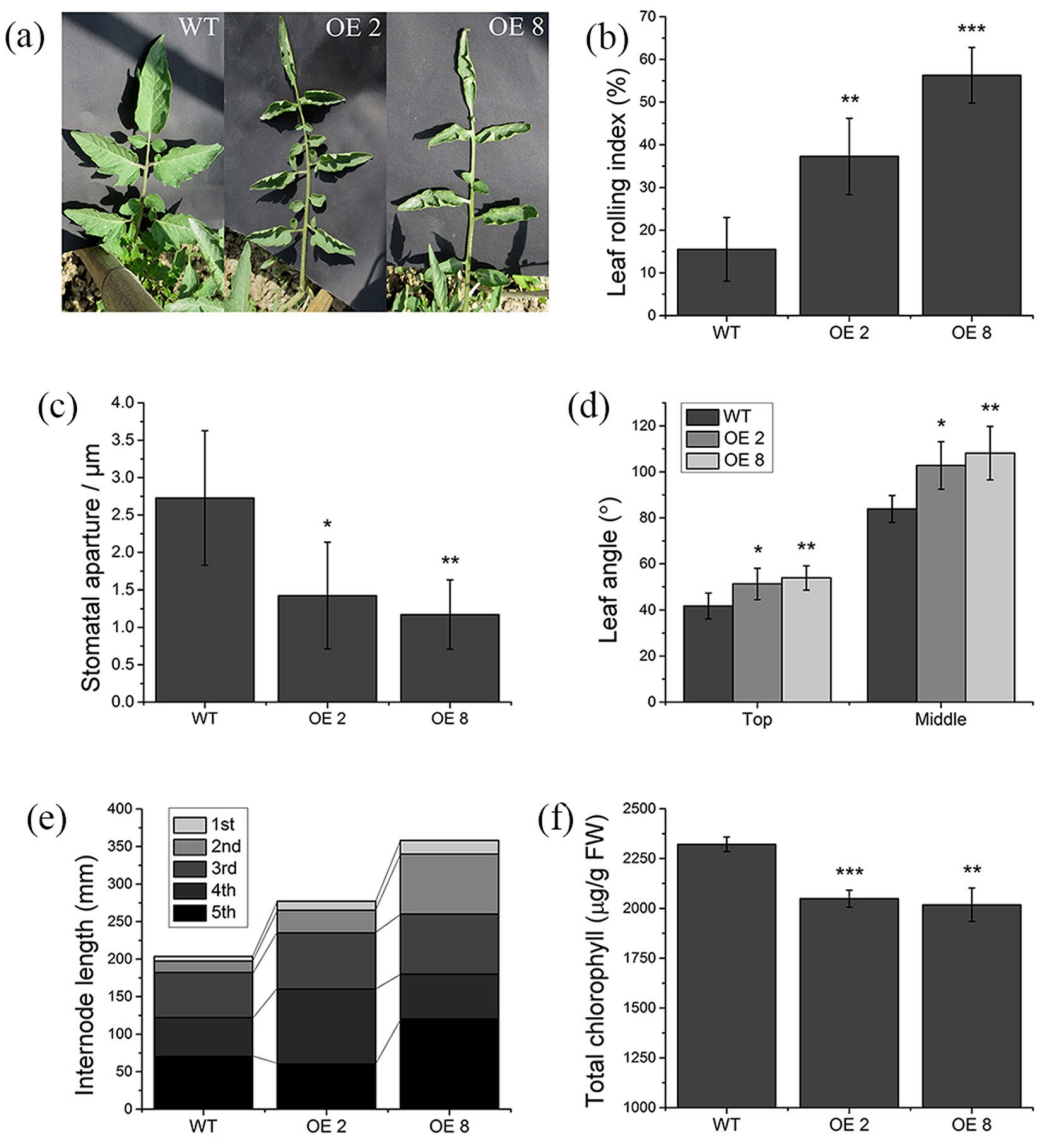
Phenotype of the *SlPRE2* overexpression transgenic plants. (**a**) Leaf phenotype of wild type and transgenic lines. The *35 S:PRE2* transgenic lines showed rolling leaf trait. (**b**) Leaf rolling index (LRI) for the mature leaves. (**c**) Stomatal aperture of mature leaves on wild type and *SlPRE2* overexpression lines. (**d**) Leaf tilt angle of the top young leaves and the middle mature leaves in wild type and transgenic lines. (**e**) The internode length for each of the top five internodes. (**f**) Chlorophyll content of leaves at the fifth from the top. Data are the mean ± SD of three biological replicates. *Significantly different from the wild type, P value *t* test < 0.05; ** for P < 0.01.

To investigate the physiological function of *SlPRE2* in tomato, transgenic lines overexpressing *SlPRE2* were generated using constitutive CaMV 35 S promoter. Expression levels of *SlPRE2* in independent *35 S:PRE2* lines were shown in Fig. S2. Compared with the wild type, the *35 S:PRE2* lines had various differences in plant morphology. First, *35 S:PRE2* lines had rolling mature leaves and the leaf rolling index was significantly increased (Fig. 2a,b, Fig. S3c), while the young leaves had no significant change (Fig. S3a,b). By scanning electron microscope analysis, the *35 S:PRE2* mature leaves had a significantly narrower stomatal aperture than that in wild type on the abaxial leaf surface, while there was no significant difference between the two groups in the stomatal pore length (Fig. 2c, Fig. S4). Furthermore, the transgenic plants had increased leaf angles and longer internodes (Fig. 2d,e). These results indicated that *SlPRE2*-overexpression affects the vegetative growth of tomato.

### Overexpression of *SlPRE2* inhibits chlorophyll accumulation and changes water losing rate in leaves

**Figure 3:**
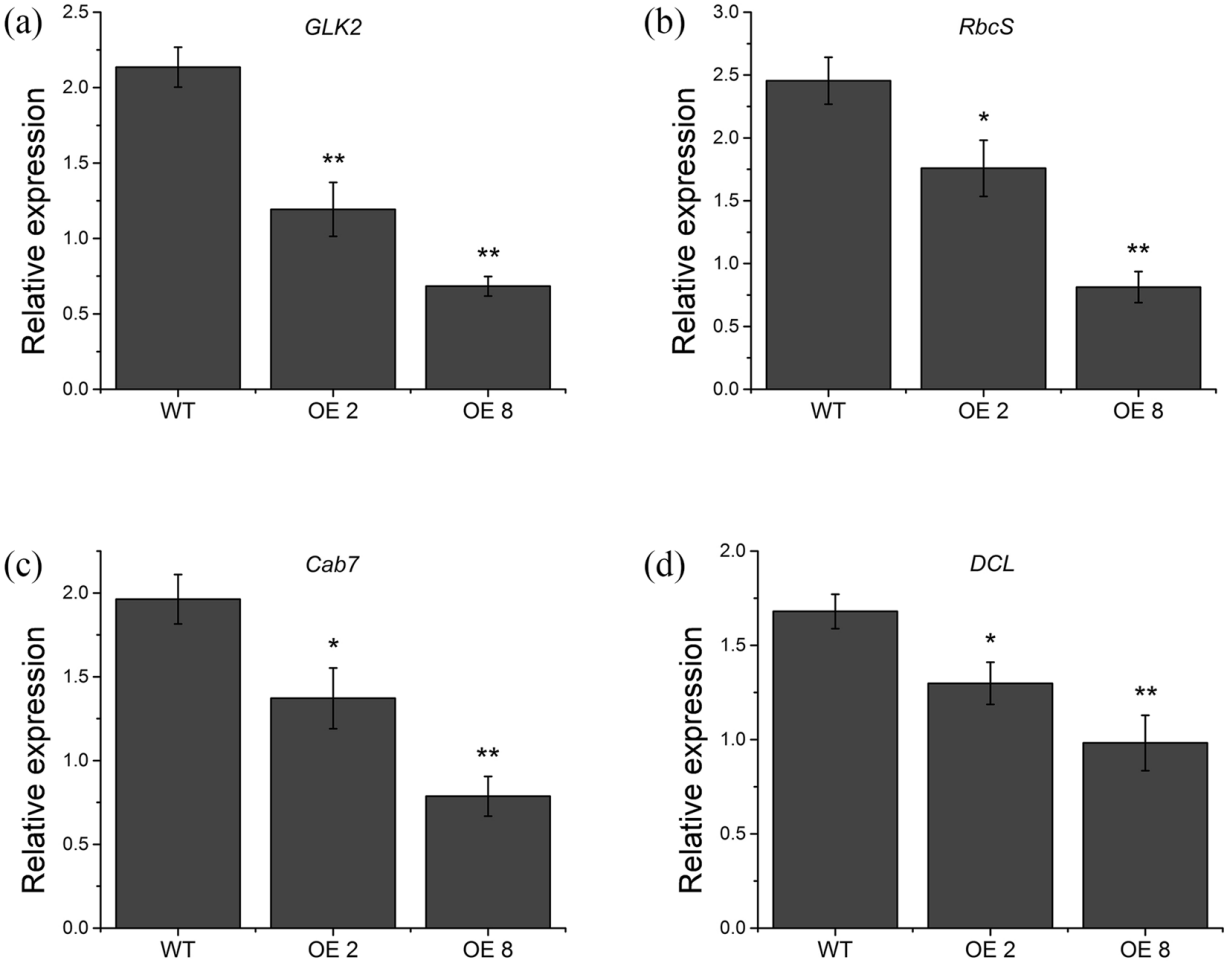
Expression levels of *GLK2*, *RbcS*, *Cab7*, and *DCL* in mature leaves of wild type and *35 S:PRE2* lines. Data are the mean ± SD of three biological replicates. *Significantly different from the wild type, P < 0.05; ** for P < 0.01.

In addition to the changes in plant morphology, the leaves of *35 S:PRE2* lines also showed light green with decreased chlorophyll content (Fig. 2f). For understanding the molecular mechanism of chlorophyll breakdown, the photosynthesis and chloroplast development-related genes, *GLK2*, *RbcS*, *Cab7*, and *DCL*, were analyzed in wild type and *35 S:PRE2* mature leaves. Quantitative RT-PCR results displayed that the four genes were significantly down-regulated in *35 S:PRE2* lines (Fig. 3).

**Figure 4:**
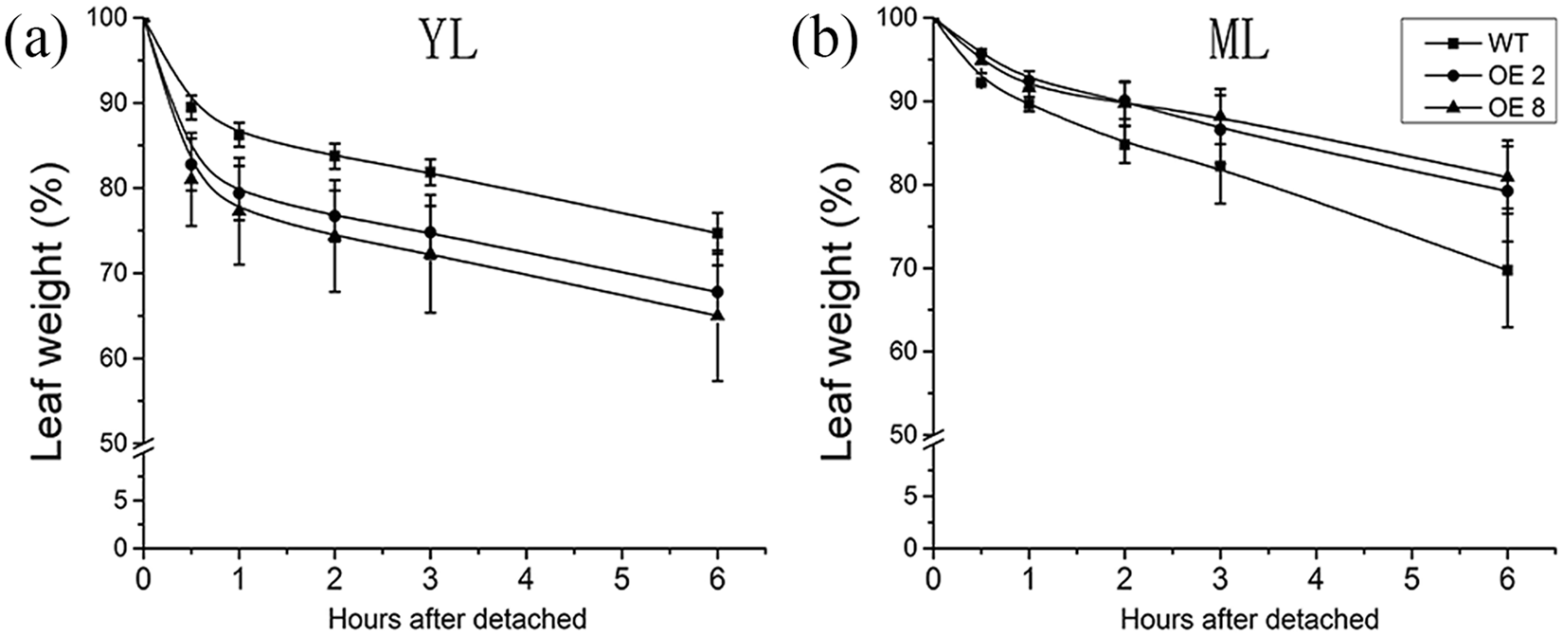
Rate of water loss from wild type and *35 S:PRE2* lines detached leaves at 25 °C room temperature. (**a**) and (**b**) represent rate of water loss in young leaves (YL) and mature leaves (ML), respectively. Data are the mean ± SD of three biological replicates.

The change of plant morphology could also affect the adaptation of plants to environment^32^. The rate of water loss of detached leaves was measured in young leaves and mature leaves from *35 S:PRE2* and wild type. As shown in Fig. 4, the young leaves of *35 S:PRE2* lines had a higher rate of water loss than wild type (Fig. 4a), however, the mature leaves of transgenic plants exhibited a lower rate of water loss (Fig. 4b).

### Overexpression of *SlPRE2* promotes hypocotyl elongation

**Figure 5:**
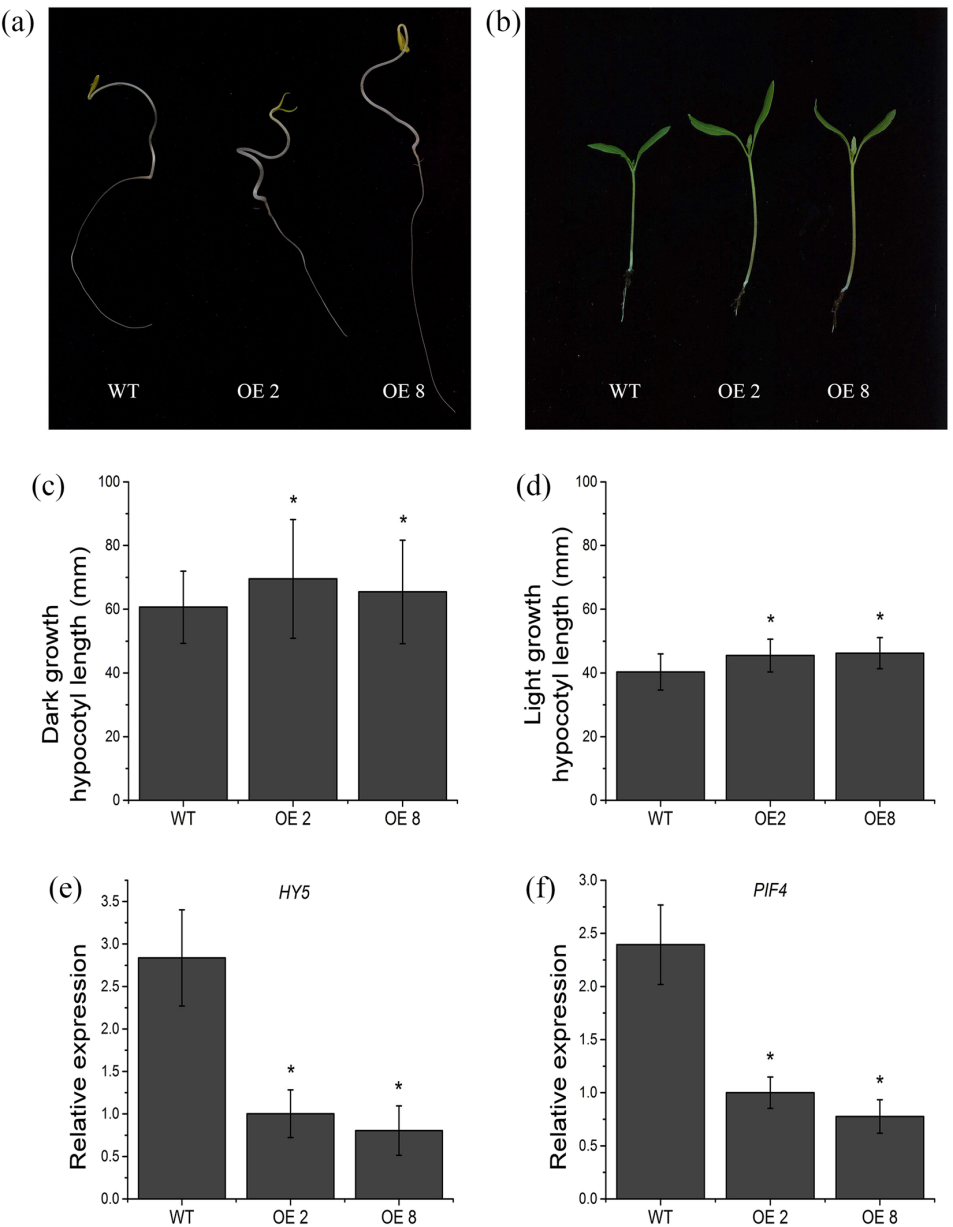
Phenotype of *35 S:PRE2* seedling and expression analysis of *HY5* and *PIF4*. (**a**) and (**b**) photograph of seedling of two *35 S:PRE2* lines and wild type obtained at 8 days after sowing under dark and light condition, respectively. (**c**) and (**d**) hypocotyl length of two *35 S:PRE2* lines and wild type 8 days seedlings growing in dark and light condition, respectively. (**e**) and (**f**) respectively represent expression levels of *HY5* and *PIF4* in 8-days-old dark-grown seedlings. Data are the mean ± SD of three biological replicates. *Significantly different from the wild type, P < 0.05; ** for P < 0.01.

To determine the roles of *SlPRE2* in morphogenesis, the measurement of hypocotyl length was performed. Seeds of the wild type and *SlPRE2* overexpression lines were germinated in dark or light for 8 days. Seedlings of transgenic lines grown in dark and light condition all showed an increase in hypocotyl growth compared with the wild type (Fig. 5a–d). To gain further information on the long hypocotyl phenotype of *35 S:PRE2* seedling, the transcription levels of *HY5* and *PIF4* were determined in 8-days-old dark-grown tomato seedlings. The *HY5* has been reported to be a light signaling related gene in *Arabidopsis* and tomato^33, 34^. The *PIF4* is a putative phytochrome interacting factor that may participate in light signaling in tomato. As shown in Fig. 5e and f, levels of *HY5* and *PIF4* mRNA were all decreased in *35 S:PRE2* seedlings.

### Overexpression of *SlPRE2* reduces pigment accumulation in fruit

**Figure 6:**
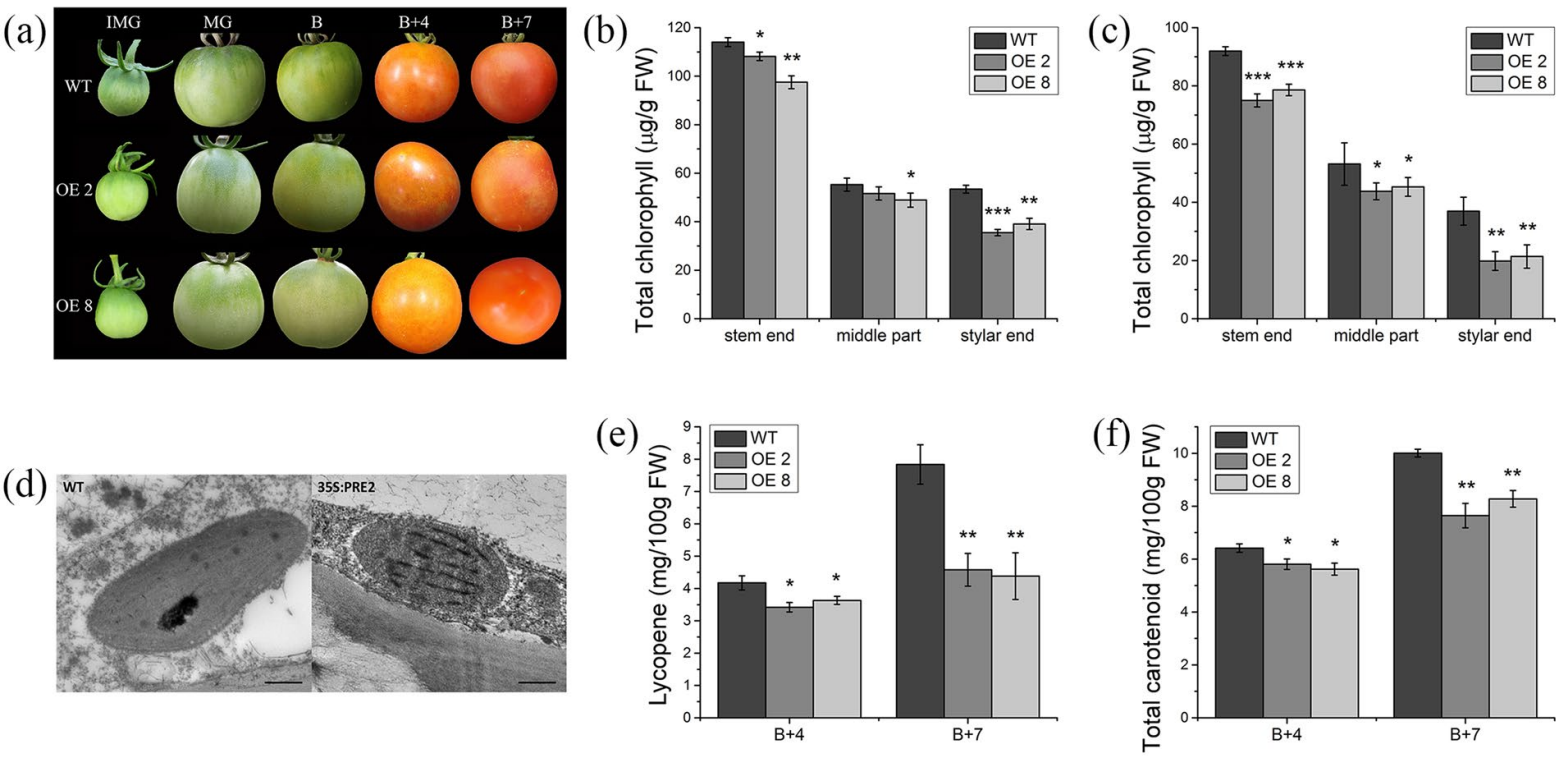
Phenotype and physiology in transgenic tomato fruits. (**a**) Fruits phenotype of wild type (top) and *35 S:PRE2* lines at IMG, MG, B, B + 4 and B + 7 stages (middle and bottom). (**b**) and (**c**) Chlorophyll contents of three latitudinal sections from wild type and *35 S:PRE2* fruits in MG and B stage. (**d**) Transmission electron microscopy images of MG fruit outer pericarp chloroplasts from wild type and *35 S:PRE2* lines. Bars = 0.5 μm. (**e**) and (**f**) Lycopene and total carotenoid content of fruit flesh in B + 4 and B + 7 stages. MG, mature fruit; B, breaker fruit; B + 4, 4 days after breaker stage; B + 7, 7 days after breaker stage. FW, fresh weight. Data are the mean ± SD of three biological replicates. *Significantly different from the wild type, P value *t* test < 0.05; ** for P < 0.01.

For most fruits, the color will change during the ripening process. Tomato fruit ripening is characterized by a shift in color from green to red, which is induced by the breakdown of chlorophyll and the formation of carotenoids. In this study, the *35 S:PRE2* fruits showed light green pericarp at IMG, MG, and B stages, and yellowing pericarp at B + 4 and B + 7 stages compared to the wild type fruits (Fig. 6a). For chlorophyll content analysis, the tomato was cut into five sections along the vertical axis. The chlorophyll contents had a gradient descent from stem end to stylar end and the transgenic fruits have less chlorophyll than the WT at MG and B stages, especially in the stylar end (Fig. 6b,c). Transmission electron microscopy (TEM) revealed that the transgenic MG fruits display smaller chloroplasts and significantly fewer grana thylakoids as well as plastoglobule per chloroplast than the wild type (Fig. 6d). These results suggested that *SlPRE2* is involved in the regulation of chloroplast development and chlorophyll accumulation in tomato. Besides, carotenoids that accumulated in ripening tomato fruit include lycopene, β-carotene, and lutein. Among them, lycopene is the main carotenoid and responsible for the red color of red tomatoes. Thus, the carotenoid contents in B + 4 and B + 7 fruits were determined by spectrophotometer. Total carotenoid contents were reduced by 10% to 25% in B + 4 and B + 7 fruits of transgenic lines (Fig. 6f). Meanwhile, the lycopene contents were decreased by 18% to 40% in transgenic B + 4 and B + 7 fruits (Fig. 6e).

### Transcriptional analysis of pigment-related genes in *35 S: SlPRE2* fruit

**Figure 7:**
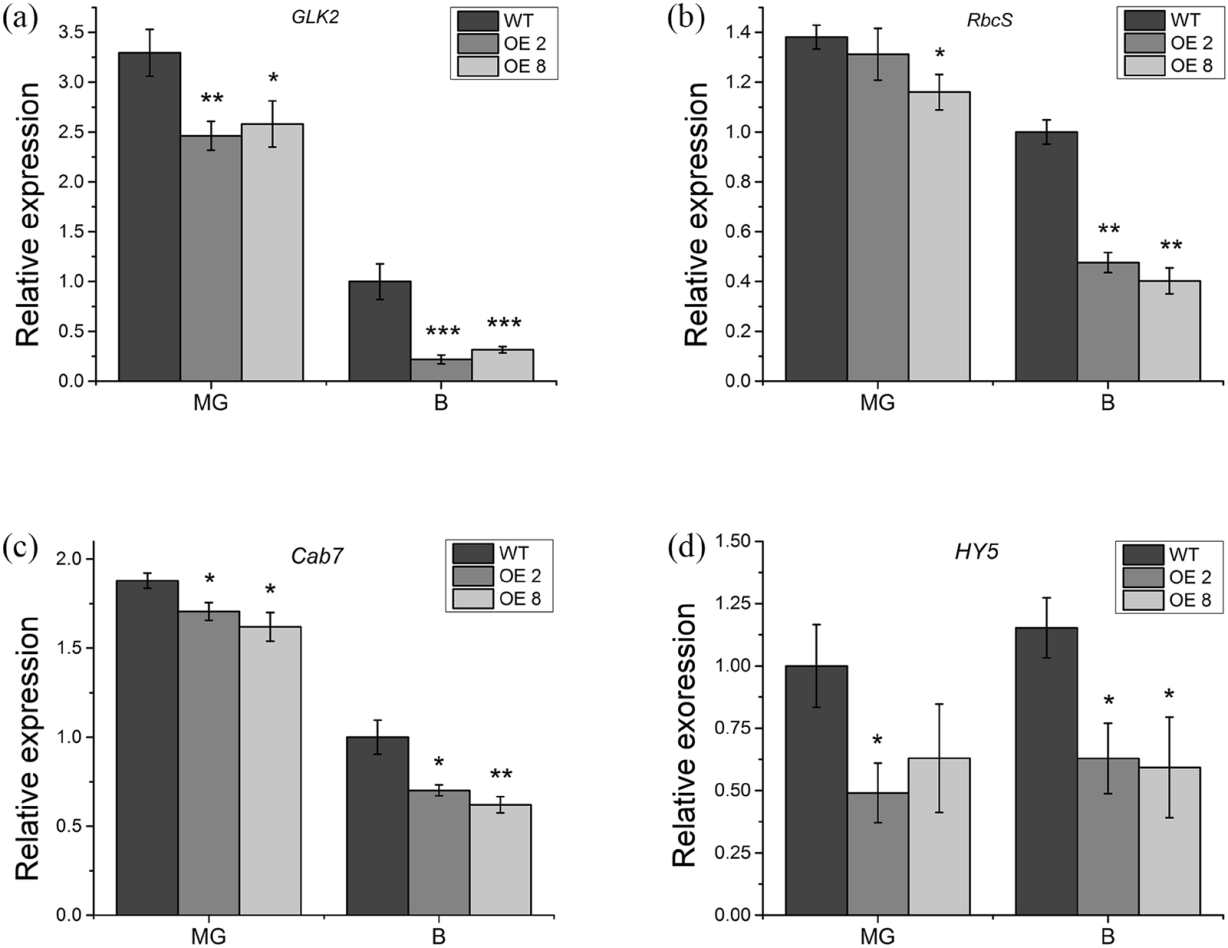
Chlorophyll metabolism related genes expression in wild type and *35 S:PRE2* lines. (**a**) to (**d**) respectively represent expression levels of *GLK2*, *RbcS, Cab7*, and *HY5* in different stage fruits of wild type and *35 S:PRE2* lines. MG, mature fruit; B, breaker fruit. Data are the mean ± SD of three biological replicates. *Significantly different from the wild type, P < 0.05; ** for P < 0.01.

**Figure 8:**
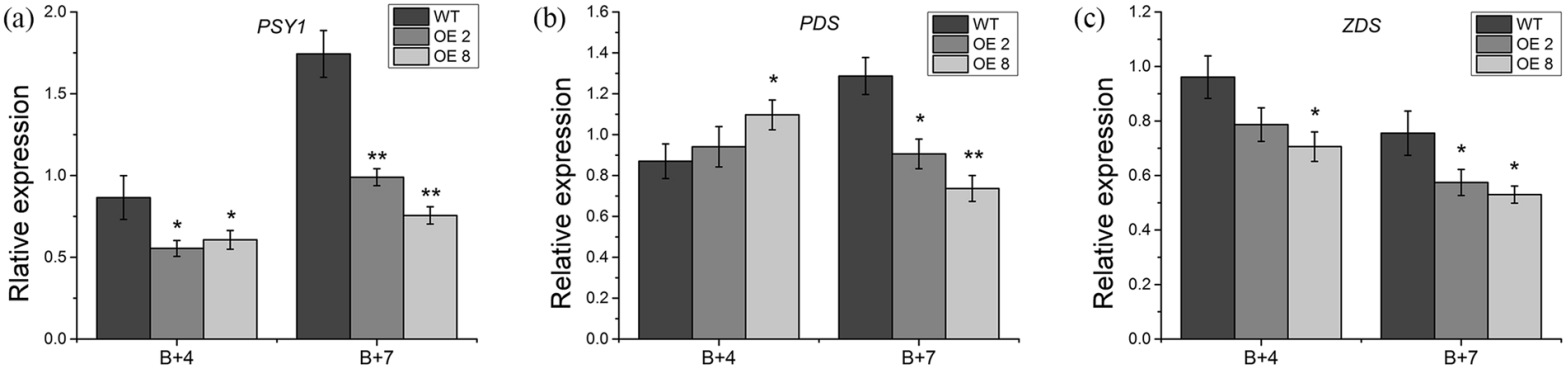
Carotenoid biosynthesis genes expression in fruits from *35 S:PRE2* lines and wild type. (**a**) to (**c**) respectively represent expression levels of *PSY1*, *PDS*, and *ZDS* in different stage fruits of wild type and *35 S:PRE2* lines. B + 4, 4 days after breaker stage; B + 7, 7 days after breaker stage. Data are the mean ± SD of three biological replicates. *Significantly different from the wild type, P < 0.05; ** for P < 0.01.

To further study the underlying causes of the decreased pigments in *35 S:PRE2* fruits, qRT-PCR was used to measure the transcript levels of genes involved in chlorophyll accumulation and carotenoid biosynthesis. The transcription levels of *GLK2*, *RbcS*, *Cab7* and *HY5* genes were notably down-regulated in *35 S:PRE2* fruits (Fig. 7). Moreover, *GLK2* expression showed a descending trend from stem end to style end, which was consistent with the previous report described by Nguyen^35^, and its transcript accumulation in transgenic fruits was lower than the wild type in MG and B (Fig. S5). In addition, the carotenoid biosynthesis genes, *PSY1* and *ZDS*, were markedly down-regulated at B + 4 and B + 7 stages, and the *PDS* was slightly increased in B + 4 fruit, but significantly decreased in B + 7 fruit (Fig. 8).

## Discussion

Light is an important factor for plant growth, which affects various development processes, such as de-etiolation, cell elongation and shade avoidance through the regulation of many genes. Previous studies have demonstrated that *PREs* involve in plant growth regulation and light signal transduction^14, 20, 25^. In *Arabidopsis*, *ATBS1/PRE3* suppresses the dwarf phenotype of BR mutants by an inhibition of negative BR signaling components, and it is involved in light signaling pathway by affecting the expression of genes related to light signaling, including *PIF3* and *HY5* ^24, 25^. With activation tagging technology, the *PRE1* activation-tagged mutant showed long hypocotyl and slightly narrow pale green leaves, and the hypocotyls length were all increased in transgenic *Arabidopsis* with overexpression of *PRE2*-*PRE5* genes^19^. Mara *et al*. showed that mutant in *PRE4/BNQ3* induced a pale-green flower and declined chlorophyll content in *Arabidopsis* ^26^. KIDARI was identified as a repressor of light signaling and photomorphogenesis in *Arabidopsis*, and co-expression of *KIDARI* suppressed *HFR1-ox* phenotypes and showed relatively long hypocotyls^20, 27^. Along with PRE1, these atypical bHLH transcription factors interact with HFR1 and PARs and rescue PIFs activity in light signaling^23^. Rice plants with overexpression of *BU1* and *ILI1* all showed increased bending of lamina joint respectively, while RNAi plants had erect leaves^21, 28^.

In our study, overexpression of *SlPRE2* resulted in various plant morphological variation. Firstly, although the shape of young leaves had no difference between the wild type and transgenic lines, the young leaves in transgenic lines had higher water loss rate than the wild type, and the mature leaves of the *35 S:PRE2* plant were curled upwardly with reduced chlorophyll content and water loss rate (Figs 2f and 4). The phenotype of pale green leaves in *35 S:PRE2* lines are consistent with the character performed in *PRE1* activation-tagged mutant leaves and *PRE4/BNQ3* mutant sepals and carpels^19, 26^. It is well known that the degree of leaf rolling are linearly related to leaf water potential and plants with curly leaves also had an increased resistance to water stress^36^. Leaf rolling reduced effective leaf area and transpiration, thus leading to the closure of stomata, which increases drought avoidance^37^. Our results revealed that overexpression of *SlPRE2* elevates the rate of water loss of young leaves. Thus, we can speculate that mature leaves rolling may be one way to hold water in leaves.

Secondly, the leaf angle and stem internode length were increased in *35 S:PRE2* lines (Fig. 2d,e), which is in line with phenotypes displayed in *ILI1-* and *BU1-*overexpression rice plants^21, 28^. Plants under shade avoidance also showed increased stem internode and leaf angle to get more light for photosynthesis^38, 39^. Given that the effective area of curly mature leaves of *35 S:PRE2* plant was decreased, this morphological variation may be a strategy for light competition by increasing effective area of leaf for receiving light and improving photosynthetic efficiency. Besides, promoter analysis of the *SlPRE2* gene showed that there are several light responsiveness cis-acting regulatory elements in the 800 bp upstream of the start codon (Table S2). Meanwhile, *SlPRE2* was significantly inhibited in strong light (Fig. 1c), and *35 S:PRE2* seedlings showed long hypocotyls along with the remarkably repressed expressions of light signaling related genes *PIF4* and *HY5* (Fig. 5e,f). These results indicated that *SlPRE2* affects plant morphology probably through influencing light signaling transduction.

Fruit ripening is a complex process, including loss of green color and accumulation of carotenoid, soften pericarp with cell wall degradation and metabolism change with flavor and nutrient accumulation. Tomato fruit ripening is influenced by multiple factors, such as development signal, phytohormones, nutrient status, temperature, and light. Among them, ethylene is a major trigger for fruit ripening in tomato. Besides, many genes affect fruit ripening through controlling chlorophyll metabolism, photosynthesis, and light signaling. For example, the *UNIFORM RIPENING* (*U*) encodes an MYB transcription factor *Golden 2-like* (*SlGLK2*) which controls chlorophyll accumulation and distribution in developing tomato fruit, and the *uniform ripening* (*u*) mutant has a light green fruit phenotype, while overexpression of *SlGLK2* results in dark-green fruits^40^. The *SlHY5* is not only an important light signaling related gene but also a positive regulator of fruit pigmentation. Down-regulation of *SlHY5* by RNA interference shows light green immature fruits with a reduced chlorophyll accumulation^34^. In addition, chlorophyll a/b-binding protein (CAB) and ribulose-1,5-bisphosphate carboxylase/oxygenase small subunit (RbcS) are reported to be essential to photosynthesis rate in photosynthetic tissues^41, 42^. In this study, overexpression of *SlPRE2* resulted in reduced chlorophyll content in unripe fruit (Fig. 6), which phenotype is consistent with that in *35 S:PRE2* leaves we identified (Fig. 2f). Moreover, the transcript levels of *GLK2*, *HY5*, *RbcS*, and *Cab7* in *35 S:PRE2* leaves and fruits were significantly decreased (Figs 3, 7). Transmission electron microscopy (TEM) showed that the transgenic MG fruits displayed defective chloroplast (Fig. 6d). Together with the expression pattern of *SlPRE2* in fruits of different period, it is possible that *SlPRE2* might regulate the balance between frequent fruit cell expansion and chlorophyll accumulation processes in immature fruit. However, these results indicated that *SlPRE2* is a repressor of chlorophyll accumulation and chloroplast development.

Carotenoids are one of the most diverse classes of natural compound. It was also accumulated in leaves, flowers, fruit of plants. They are essential for plant photosynthesis and protection cell from photo-oxidation. Moreover, they furnish flowers and fruits with distinct colors that are designed to attract animals to disperse seeds. So far, the carotenoid biosynthesis pathway is very clear in higher plant. Generally, the first dedicated compound in the carotenoid pathway, 15-*cis*-phytoene, is formed through the catalyzes of phytoene synthase1 (PSY1), a key enzyme for carotenoid biosynthetic pathway^43^. After that, phytoene desaturase (PDS) and ζ-carotene desaturase (ZDS) catalyze the synthesis of 9,9′-di-*cis*-ζ-carotene and all-*trans*-lycopene^44, 45^. Absent function of PSY1 in tomato fruit results in yellow flesh phenotype^43^. In this study, we analyzed the expression of these tomato genes, *PSY1*, *PDS*, and *ZDS*. qPCR revealed that the transcript levels of these genes were significantly reduced in ripe fruits, especially in B + 7 transgenic fruits (Fig. 8), which were confirmed by the reduced lycopene and total carotenoid contents in *35 S:PRE2* fruits (Fig. 6e,f). These results suggested that *SlPRE2* negatively regulates carotenoid accumulation during fruit ripening by repressing the expression of these carotenoid biosynthetic genes.

## Materials and Methods

### Plant materials and growth conditions

In this study, the tomato (*Solanum lycopersicum* Mill. cv. Ailsa Craig) was used as the wild type. Plants were grown in standard greenhouses with 16 h light (27 °C) and 8 h dark (19 °C) cycle. In wild type, the developmental stages of tomato fruits were divided into IMG (immature green at 15 DPA), MG (mature green at 32DPA), B (breaker with the color change from green to yellow), B + 4 (4 days after breaker) and B + 7 (7 days after breaker) stages according to the days post anthesis (DPA) and fruit color. For a test of gene expression under different light condition, plants were exposed in weak (50 μmol·m^−2^·s^−1^) or strong (800 μmol·m^−2^·s^−1^) light condition for 8 hours, respectively. All plant samples were immediately frozen in liquid nitrogen and stored at −80 °C.

### Multiple sequence alignment analysis

Multiple sequence alignment of *SlPRE2* with other *PRE*-like protein was conducted using the ClustalX 1.83 and DNAMAN version 7.0 programs. The peptide sequences were selected from *Arabidopsis thaliana, Solanum lycopersicum* and *Oryza sativa* according to their sequence similarity and functions reported. For phylogenetic analysis, peptide sequences of PREs proteins were selected, and the phylogenetic tree was constructed using MEGA 5 software.

### Total RNA isolation and quantitative real-time PCR analysis

Total RNA was extracted from various tissues using RNAiso Plus (Takara). 2 μg RNA was treated with DNase I (Promega). The first-strand cDNA synthesis was performed with the M-MLV reverse transcriptase (Promega) using oligo(dT)_20_ primer. The synthesized cDNA was diluted three times with nuclease-free water for qRT-PCR analysis. Quantitative real-time PCR was performed using the GoTaq qPCR Master Mix (Promega). The condition for qPCR amplification was as follow: 95 °C for 3 min; 40 cycles of 95 °C for 30 s, 60 °C for 45 s and 72 °C for 30 s. Amplification was followed by a melting curve analysis with continual fluorescence data acquisition during the 60–95 °C melt. *SlCAC* and *SlEF1α* were used as internal reference genes for tomato development and abiotic stress studies, respectively^46, 47^. In addition, gene-specific primers for qPCR analysis are listed in Table S3.

### Vector construction and plant transformation

To generate *35 S:PRE2* lines, the full-length coding sequence of the *SlPRE2* gene was amplified by PCR from tomato (AC) cDNA using primers SlPRE2-F (5′ CGGGATCCTCAAAAGAATCATCTCAAAATA 3′) and SlPRE2-R (5′ CGAGCTCGTAAACATCAATACAAGCACAC 3′) which have been tailed with *Bam*H I and *Sac* I restriction site at the 5′ end respectively. The amplified products were digested and linked into the plant binary vector pBI121 to produce the *SlPRE2-*overexpression vector pBI121-*SlPRE2* driven by the CaMV 35 S promoter. After that, the pBI121-*SlPRE2* vector was transferred into tomato cotyledon explants through *Agrobacterium*-mediated transformation method as described by Chen *et al*.^48^. The transgenic plants were selected on kanamycin medium and detected by PCR with primers NPT II-F (5′ GACAATCGGCTGCTCTGA 3′) and NPT II-R (5′ AACTCCAGCATGAGATCC 3′).

### Leaf rolling index and leaf angle measurements

The measurements of leaf rolling index and leaf angle were taken during the 9:00 to 11:00 AM using ruler and protractor respectively. The natural width (Ln) and the greatest width (Lg) of the leaf blade were determined. Leaf rolling index was calculated according to the following equation^49^: Leaf rolling index (%) = (Lg − Ln)/Lg × 100. Leaf angle was determined as the adaxial angle between a stem and a leaf ^50^.

### Chlorophyll and carotenoid analysis

Chlorophyll contents were measured in the expanded leaves (mature leaves), IMG (25DPA), MG and B fruits. Tissues from the IMG, MG and B fruits were divided into five latitudinal sections along the vertical axis, and the stem end, middle part and stylar end were selected to determine the chlorophyll contents respectively^35^. The selected tissues (~0.5 g) were ground and dissolved in 10 mL of 80% acetone. The extract was centrifuged at 1500 g for 10 min, then the supernatant was transferred to a new lightproof tube, and its absorbance was measured at 645 nm and 663 nm with a spectrophotometer (lambda 900 UV/VIS/NIR, Perkin Elmer). Total chlorophyll contents were calculated with the following equation: total chlorophyll (mg/l FW) = 8.02A663 + 20.2A645^51, 52^. Carotenoids were measured in the B + 4 and B + 7 fruit. The selected tissues (~1.0 g) were ground and dissolved in 10 mL of 60:40 (v/v) hexane: acetone. The extract was centrifuged at 4000 g for 5 min and the absorbance of the supernatant was measured at 450 nm with a spectrophotometer. Total carotenoid contents were calculated with the following equation: total carotenoids (mg/100 g FW) = 4 × A450 × 10^43, 53^. For lycopene quantitation, 0.25 g pericarps from the B + 4 and B + 7 fruits were ground and dissolved in 8 mL of 2:1:1 (v/v/v) hexane: ethanol: acetone. The extract was shaken for 30 min then 1 mL distilled water was added and shaken for 5 minutes. The extracted solution was left for 15 min to separate into two phases (polar upper layer and nonpolar layer). The absorbance of the supernatant was measured at 503 nm by spectrophotometer. Lycopene contents were quantified using a molar extinction coefficient of 1.585 × 10^5^ M^−1 ^cm^−1^ ^54^.

### Transmission electron microscopy and scanning electron microscopy

Outer pericarp tissues from MG fruits were selected and fixed in 2.5% glutaraldehyde in 0.1 M phosphate buffer for 4 h and washed three times in the 0.1 M phosphate buffer for 15 min. Then tissues were postfixed in 1% OsO_4_ at 4 °C for 1 hour, and dehydrated in an ethanol series and infiltrated in spur resin. Ultrathin sections were viewed with Hitachi H-7500 transmission electron microscope. For analysis of stomatal characteristics, mature leaves of wild type and transgenic plants were fixed in 2.5% glutaraldehyde. After being fixed for 3 hours, leaf tissues were dehydrated through an ethanol series. Then the samples were dried and coated with gold for observation using Hitachi scanning electron microscope S-3000N. The stomatal size was measured using Image J software.

### Statistical analysis

Data were analyzed by one-way analysis of variance (ANOVA) and the t-test. Differences were considered to be significant at a level of p < 0.05. The measurement values were displayed as means with standard deviation (SD) of three biological replicates.

## Electronic supplementary material

Supplementary information
