# Supplementary material for: Overexpression of SlPRE2, an atypical bHLH transcription factor, affects plant morphology and fruit pigment accumulation in tomato
Source: Sci Rep. 2017 Jul 19;7:5786. doi: 10.1038/s41598-017-04092-y (PMC5517572; doi:10.1038/s41598-017-04092-y)
Supplement: Supplementary file 1 — Supplementary information [file 41598_2017_4092_MOESM1_ESM.pdf]

Supplementary Information

Overexpression of *SlPRE2*, an atypical bHLH transcription factor, affects plant morphology and fruit pigment accumulation in tomato

Zhiguo Zhu, Guoping Chen, Xuhu Guo, Wencheng Yin, Xiaohui Yu, Jingtao Hu, Zongli Hu*
Laboratory of molecular biology of tomato, Bioengineering College, Chongqing University, Chongqing 400044, People's Republic of China

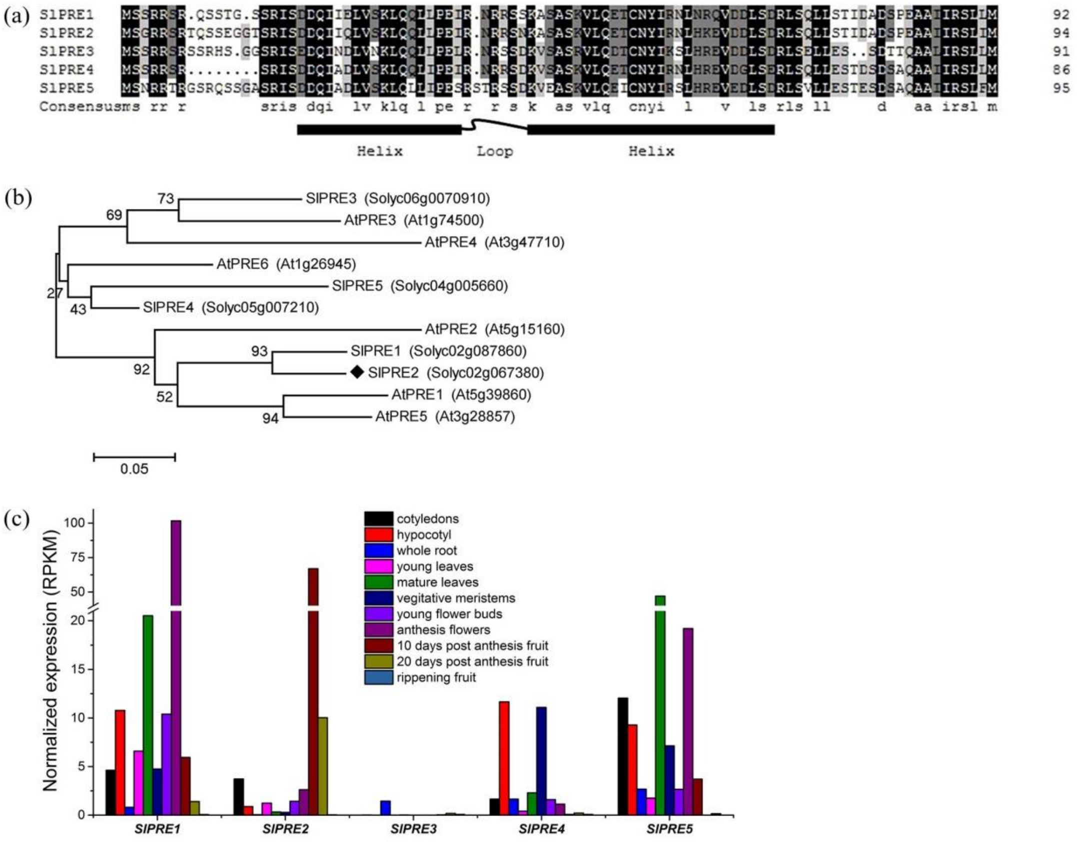

Figure S1. Sequence analysis of SlPRE2. (a) Multiple sequence alignment of SlPRE2 with other bHLH proteins in tomato. Black and gray backgrounds indicate identical and similar amino acids. Convergence in structure is indicated by black box and curve. SlPRE1/Slstyle2.1, Solyc02g087860. SlPRE2, Solyc02g067380. SlPRE3/bHLH103, Solyc06g070910. SlPRE4/bHLH131, Solyc05g007210. SlPRE5, Solyc04g005660. (b) Phylogenetic tree analysis of SlPREs and PREs protein sequences in Arabidopsis. The phylogenetic tree was constructed using MEGA 5 software and the Neighbor-joining method was employed with bootstrap analysis of 1000 replicate. SlPRE2 was marked with a black diamond. (c) Transcriptome analysis of 5 SlPRE genes during 11 development stages or tissues according to SGN database.

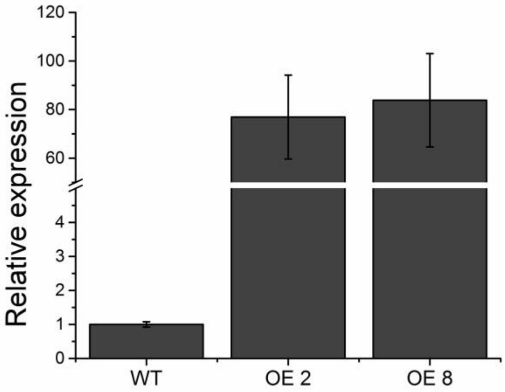

Figure S2. Relative mRNA levels of *SlPRE2* in *35S:PRE2* transgenic young leaves. Data are the mean ± SD of three biological replicates.

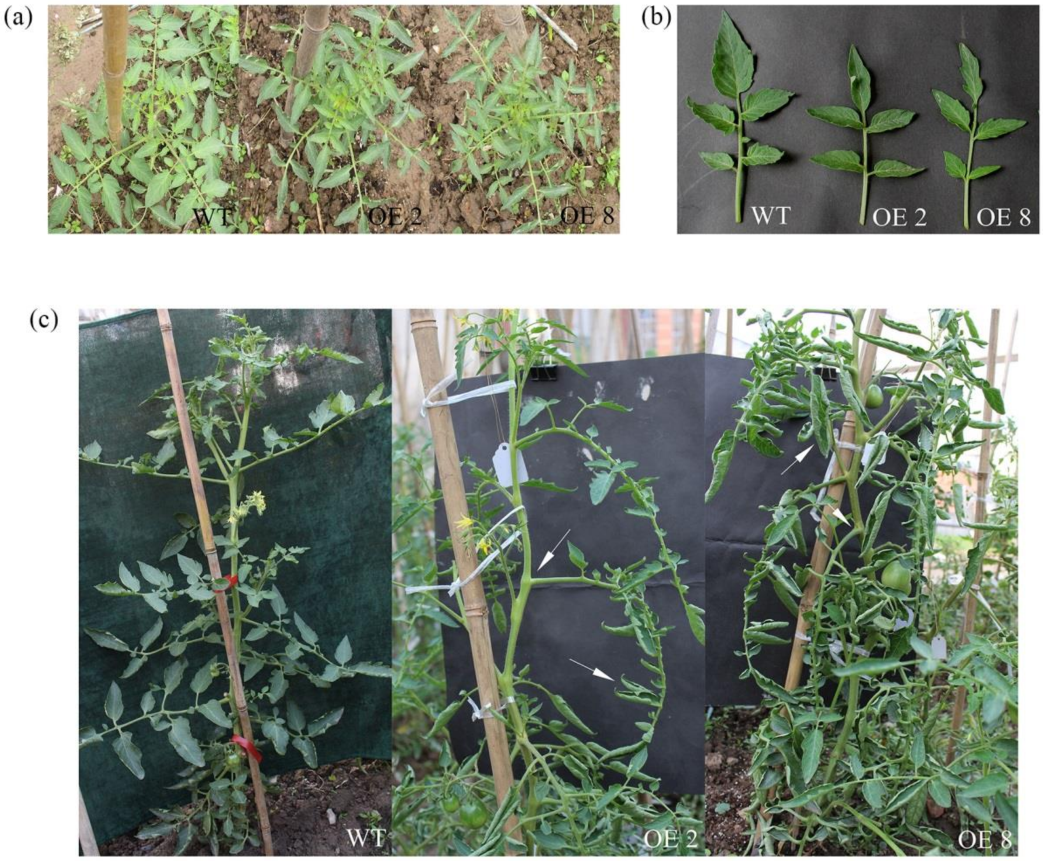

Figure S3. Phenotype of PRE2 overexpression plants. (a) and (b) respectively represent whole plant and the young leaves of 5 weeks old wild type and *SlPRE2* overexpression plants. (c) Gross morphologies of *SlPRE2* overexpression plants in the wild-type background. The white arrows indicates increased leaf angle or rolling index.

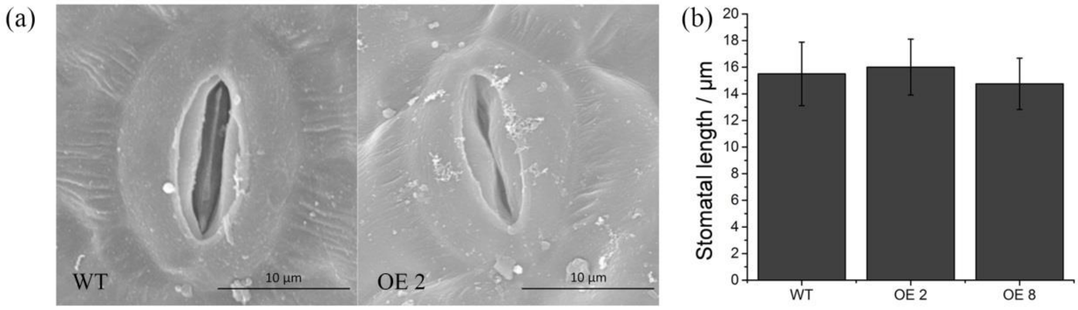

Figure S4. Stomatal aperture analysis. (a) representative status of stomata from wild type and *SlPRE2* overexpression mature leaves. (b) stomatal length in wild type and *SlPRE2* overexpression mature leaves. Data are the mean ±SD of three biological replicates.

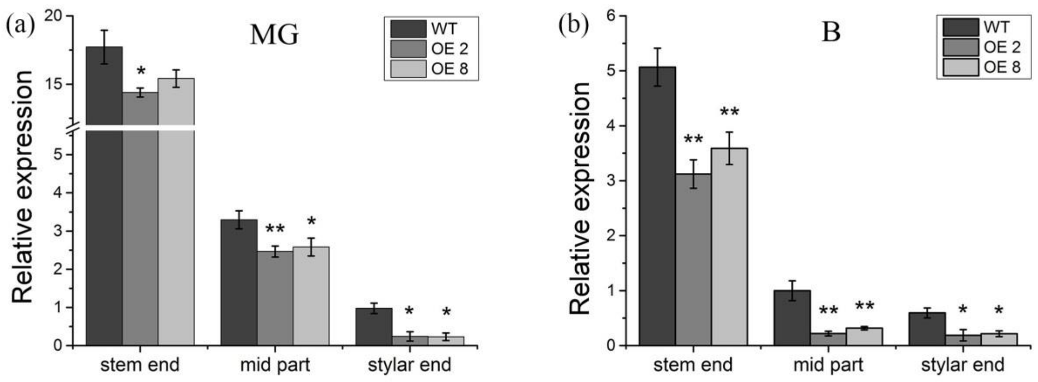

Figure S5. Gradient expression of *GLK2* in different stage fruits of wild type and *35S:PRE2* lines. (a) and (b) respectively represent gradient expression of *GLK2* in MG and B stage fruit. The *GLK2* expression levels were gradiently decreased from stem end to stylar end of the fruit and down-regulated in *35S:PRE2* lines compared with wild type. MG, mature fruit; B, breaker fruit. Data are the mean ± SD of three biological replicates. * significantly different from the wild type, $P<0.05$; ** for $P<0.01$.

Table S1. Transcriptome analysis of SlPRE2 in various *S. pimpinellifolium* LA1589 tissues using Tomato Functional Genomics Database.

Sample	Normalized expression (RPKM)
cotyledons	3.72
hypocotyl	0.88
whole root	0.04
vegetative meristems	0.28
young leaves	1.23
mature leaves	0.33
young flower buds	1.42
anthesis flowers	2.62
10 days post anthesis fruit	66.93
20 days post anthesis fruit	10.03
ripening fruit	0.03

Table S2. Analysis of light response motifs from *SlPRE2* promoter. Only the first 800 bases upstream of the initiation codon (ATG) from SGN (Sol Genomics Network) was analyzed by PlantCARE. Motifs involved in light response are marked in bold font.

Motif name	Sequence	Function	Number
**G-Box**	**CACGTT/A**	**Cis-acting regulatory element involved in light responsiveness**	**5**
**ACE**	**AAAACGTTTA**	**Cis-acting element involved in light responsiveness**	**1**
**Box 4**	**ATTAAT**	**Part of a conserved DNA module involved in light responsiveness**	**1**
**Box I**	**TTTCAAA**	**Light responsive element**	**1**
**GAG-motif**	**AGAGAGT**	**Part of a light responsive element**	**1**
**GATA-motif**	**GATAGGA**	**Part of a light responsive element**	**1**
**GT1-motif**	**GGTTAA**	**Light responsive element**	**1**
**3-AF1 binding site**	**AAGAGATATTT**	**Light responsive element**	**1**
ABRE	TACGTG	Cis-acting element involved in the abscisic acid responsiveness	2
HSE	AAAAAATTTC	Cis-acting element involved in heat stress responsiveness	2
EIRE	TTCGACC	Elicitor-responsive element	1
LTR	CCGAAA	Cis-acting element involved in low-temperature responsiveness	1
MBS	TAACTG	MYB binding site involved in drought-inducibility	1
O2-site	GATGATGTGG	Cis-acting regulatory element involved in zein metabolism regulation	1
Skn-1_motif	GTCAT	Cis-acting regulatory element required for endosperm expression	1
TCA-element	CAGAAAAGGA	Cis-acting element involved in salicylic acid responsiveness	1
circadian	CAANNNNATC	Cis-acting regulatory element involved in circadian control	1

Table S3. Primers used for quantitative RT-PCR analysis.

**Gene**	**Forward primer (5'→3')**	**Reverse primer (5'→3')**	**PCR Product length (bp)**
EF1α	TACTGGTGGTTTTGAAGCTG	AACTTCCTTCACGATTTCATCATA	166
CAC	CCTCCGTTGTGATGTAACTGG	ATTGGTGGAAAGTAACATCATCG	173
PRE2	TATGTCTGGGAGAAGGTCAAGGA	CGACGATTACGAATTTCAGGAAG	123
PSY1	AGAGGTGGTGGAAAGCAA	TCTCGGGAGTCATTAGCAT	298
PDS	GCTTTACCCGCTCCTTTA	ACCTTGCTTTCTCATCCA	174
ZDS	GGTGGGTGCTGAAAAAAAT	GGAAAGCGGAAATCAAGTT	93
DCL	CCGCAAGGATGGTGAAACA	TCCGCTTCCGAAAATGCC	116
GLK2	ACAATCGGAGGCGGAGGA	CAAGGAGTGCCTGGTACAAGAG	185
RbcS	TGCTCAGCGAAATTGAGTACCTAT	AACTTCCACATGGTCCAGTATCTG	139
Cab7	TAGACTTGCTATGTTAGCCGTTATG	TTCTGCTTCTCACTTGGGACTG	145
HY5	TGCTAGTTTCGGGTGGATTG	CTAGGGAACGCTAGCAAAGG	200
PIF4	AGTGAAACAACGGCCACAGA	TGTGGAGCAGGAAGTTCAGTC	198
